# Immunotherapy of invasive fungal infection in hematopoietic stem cell transplant recipients

**DOI:** 10.3389/fonc.2013.00017

**Published:** 2013-02-07

**Authors:** Thomas Lehrnbecher, Stanislaw Schmidt, Lars Tramsen, Thomas Klingebiel

**Affiliations:** Pediatric Hematology and Oncology, Children’s Hospital, Johann Wolfgang Goethe UniversityFrankfurt, Germany

**Keywords:** invasive fungal infection, allogeneic hematopoietic stem cell transplantation, immunotherapy, granulocyte, T cell, natural killer cell

## Abstract

Despite the availability of new antifungal compounds, invasive fungal infection remains a significant cause of morbidity and mortality in children and adults undergoing allogeneic hematopoietic stem cell transplantation (HSCT). Allogeneic HSCT recipients suffer from a long lasting defect of different arms of the immune system, which increases the risk for and deteriorates the prognosis of invasive fungal infections. In turn, advances in understanding these immune deficits have resulted in promising strategies to enhance or restore critical immune functions in allogeneic HSCT recipients. Potential approaches include the administration of granulocytes, since neutropenia is the single most important risk factor for invasive fungal infection, and preliminary clinical results suggest a benefit of adoptively transferred donor-derived antifungal T cells. *In vitro* data and animal studies demonstrate an antifungal effect of natural killer cells, but clinical data are lacking to date. This review summarizes and critically discusses the available data of immunotherapeutic strategies in allogeneic HSCT recipients suffering from invasive fungal infection.

Invasive fungal infection remains a significant cause of morbidity and mortality in children and adults undergoing allogeneic hematopoietic stem cell transplantation (HSCT). The most common fungal pathogens identified in this patient population are *Candida* spp. and in particular *Aspergillus* spp.; in the latter group, the majority of invasive infections are due to *A. fumigatus*, followed by *A. flavus*, *A. niger*, and *A. versicolor* (Morgan et al., 2005; Pagano et al., 2007). In addition, recent studies report on an increasing incidence of infections due to Mucormycetes which are usually caused by the genera *Rhizopus*, *Rhizomucor*, *Mucor*, and *Lichtheimia* (Cuenca-Estrella et al., 2009). Although the definitive diagnosis of invasive fungal infection in the population of allogeneic HSCT recipients is difficult to establish, two recent studies report on an overall incidence of 3.4 and 7.8% of proven and probable invasive fungal infections, respectively; however, it is important to note that a considerable variability across institutions has been reported, and incidence rates may vary between 0.9 and 13.2% (Dvorak et al., 2005; Pagano et al., 2007; Kontoyiannis et al., 2010).

Despite the availability of new antifungal compounds such as broad-spectrum triazoles or the new class of echinocandins, morbidity and mortality of invasive fungal infections are still unacceptably high. In this respect, recent analyses report on mortality rates between 36% (combined analysis for autologous and allogeneic HSCT) and 67% (analysis for allogeneic HSCT from unrelated donors) due to invasive aspergillosis (Mikulska et al., 2009; Neofytos et al., 2009). In patients with mucormycosis, 1-year mortality exceeded 70% (Dvorak et al., 2005; Pagano et al., 2007; Kontoyiannis et al., 2010).

Although pharmacological prophylactic measures (e.g., the institution of antifungal prophylaxis with posaconazole) and improved therapeutic approaches have decreased morbidity and mortality of invasive fungal infections, experts agree that the integrity of host defenses remains the mainstay of defense. Allogeneic HSCT is characterized by a subsequent period of immunodeficiency. The duration and severity of these defects of host immunity vary due to multiple factors, such as graft manipulation and choice of graft type (donor and source), intensity of conditioning, *in vivo* T cell depletion, and choice, duration, and intensity of pharmacological graft-versus-host-disease (GvHD) prophylaxis and treatment. The conditioning chemotherapeutic regimen, which often includes radiation, results in the disruption of mucosal barriers and eradicates the recipient’s neutrophils and lymphoid tissue. Whereas mucositis and neutropenia resolve over the first weeks after transplantation, the impairment of cellular immunity may persist over a prolonged period of time. After the recipient’s immune system is restored, the risk or the occurrence of GvHD may further induce immunologic compromises. It is well known that the impairment of these arms of the immune system significantly increases the risk for and deteriorates the outcome of invasive fungal infection. In turn, advances in understanding of these immune deficits in the allogeneic HSCT recipient have resulted in promising strategies to enhance or restore critical immune functions in order to combat invasive fungal infections, which will be critically discussed in this article.

## THE IMPACT OF GRANULOCYTES IN INVASIVE FUNGAL INFECTION: IS THERE A BENEFIT OF GRANULOCYTE TRANSFUSION?

Neutropenia is the single most important risk factor for invasive fungal infection, with the highest risk in patients with severe (absolute neutrophil count, ANC < 500/μl) and prolonged (>10 days) neutropenia. In the transplant setting, about one-third of invasive fungal infections occur in the pre-engraftment period (Bow, 2009); notably, the time to neutrophil engraftment depends on a number of factors such as conditioning regimen, stem cell source, and processing of the stem cell product. Therefore, the transfusion of granulocytes from healthy individuals has been considered as an option for adjunctive immunotherapy in patients with neutropenia or impaired neutrophil function who are at high risk for or suffer from invasive fungal infection. A retrospective case–control study in 29 cancer patients suffering from *Candida* species bloodstream infections suggested that therapy with granulocyte transfusions was associated with better than expected survival rates (Safdar et al., 2004). These data corroborated smaller case series in neutropenic children and adults with various severe infectious complications including invasive fungal infection (Mousset et al., 2005; Grigull et al., 2006; Atay et al., 2011). Although the studies described granulocyte transfusions as safe, fever and chills may be seen in up to 20% of transfusions, and pulmonary reactions (acute respiratory distress syndrome, ARDS) and human leukocyte antigen (HLA) alloimmunization have been reported as potential major complications (O’Donghaile et al., 2012). In addition, a randomized phase III study evaluating the effect of granulocyte transfusions in 74 neutropenic patients, most of them after allogeneic HSCT (*n* = 39), failed to demonstrate a significant benefit of this approach (Seidel et al., 2008). The administration of granulocytes did not improve survival rates until day 100 in patients with fungal (*n* = 55) and other infections. The authors attributed the lack of efficacy to procedural obstacles, which is supported by the observation that neutrophil reconstitution was equal in the treatment and control arm indicating that the granulocyte transfusions administered at the schedule and dose used were not sufficient to accelerate a lasting peripheral blood neutrophil reconstitution. Therefore, the results of large prospective randomized trials assessing the effect of high dose granulocyte transfusion in neutropenic patients, such as the ring study (NCT00627393) are urgently needed to prove or refute the empirical evidence of the benefit of granulocyte transfusion in this setting.

### T CELLS AND INVASIVE FUNGAL INFECTION

In contrast to a relatively rapid recovery of cells of innate immunity such as granulocytes, both the absolute levels and function of B and T lymphocytes remain abnormal for many months (Eyrich et al., 2001; Peggs and Mackinnon, 2004). T cells are known to possess some graft-versus-malignancy effect and to play an important role in the defense against viral pathogens (Bader et al., 2004; Peggs and Mackinnon, 2004). However, there is a growing body of evidence suggesting that adaptive immunity, in particular CD4^+^ T lymphocytes, has an important function in the host response against fungi. For example, non-neutropenic patients with advanced AIDS have a high risk for invasive aspergillosis (Denning, 1998). In addition, in the transplant setting, the median time of invasive aspergillosis, invasive candidiasis, and zygomycosis is 82 days (range 3–6542 days), 77 days (range 0–2219 days), and 173 days (range 7–2254 days) after HSCT, respectively, a time, when neutropenia and mucositis have generally resolved, but adaptive immune responses are still hampered (Hamza et al., 2004; Neofytos et al., 2009). An early study demonstrated that the presence of CD4^+^ T lymphocytes, which secrete interferon-gamma (IFN-γ) upon stimulation with *A. fumigatus* antigens indicating a T helper type 1 (T_H_1) cell response, correlated with a favorable outcome (Hebart et al., 2002). It has also been shown that for up to 1 year after HSCT, transplant recipients have a significantly reduced number of functionally active anti-*Aspergillus* T cells compared to healthy controls, and lymphopenia is associated with a higher risk of death of transplant recipients with invasive fungal infection (Beck et al., 2008; Mikulska et al., 2009).These data provide the rationale of adoptively transferring *in vitro* manufactured anti-*Aspergillus* T cells for prevention or for treatment of *Aspergillus* infections. In the first clinical trial, ten haploidentical transplant recipients with evidence of invasive aspergillosis (e.g., pulmonary infiltrates, positive galactomannan test) received anti-*Aspergillus* T cells in the dose range of 1 × 10^5^ to 1 × 10^6^ per kg body weight between 17 and 37 days after transplantation (Perruccio et al., 2005). In all 10 patients, galactomannan positivity disappeared within 6 weeks of infusion of anti-*Aspergillus* T cells whereas it persisted in the 13 controls not receiving immunotherapy for as long as monitored. In addition, 9 out of the 10 patients who had received anti-*Aspergillus* T cells cleared invasive aspergillosis, whereas this was seen in only 7 out of the 13 controls. Anti-*Aspergillus* T cell clones were prepared by using heat-inactivated conidia for stimulation followed by limiting dilution which involved complex cell manipulation.

Consequently, there was much interest in improving the techniques for the clinical scale-generation of anti-*Aspergillus* T cells. Tramsen et al. (2009) used an *Aspergillus* extract for stimulation; pathogen-specific antifungal cells were selected by means of the IFN-γ secretion assay and expanded. Out of a total of 1.1 × 10^9^ white blood cells from a leukapheresis product, a median number of 2 × 10^7^ CD4^+^ T cells were obtained within 13 days. The cultured anti-*Aspergillus* cells exhibited almost exclusively a memory activated T_H_ cell phenotype. Upon re-stimulation, the generated cells produced IFN-γ, but not interleukin (IL)-4 or IL-10, indicating a T_H_1 population. In addition, the cells were not end-differentiated T cells, since they proliferated upon re-stimulation. Interestingly, the functionally active anti-*Aspergillus* T_H_1 cells elicited limited cross-reactivity with other *Aspergillus* species and fungi (Beck et al., 2006). As compared to unselected CD4^+^ cells, the generated anti-*Aspergillus* T cells exhibited reduced alloreactivity *in vitro* (Tramsen et al., 2009).

Recently, a simple, robust and clinically applicable procedure of generating anti-*Aspergillus* T cells was reported using an environmental strain of *A. fumigatus* and materials and reagents for clinical manufacture (Gaundar et al., 2012). In this protocol, cells were expanded with a cocktail of IL-2, IL-7, and IL-15, which resulted in a 30-fold increase in cell numbers over 21 days of culture. Generated cells were predominantly effector and central memory CD4^+^ T cells, which produced T_H_1 and T_H_17 cytokines and expanded upon re-stimulation.

Although most groups have employed lysates from *Aspergillus* isolates for the generation of protecting anti-*Aspergillus* T cells (Perruccio et al., 2005; Tramsen et al., 2009; Khanna et al., 2011; Gaundar et al., 2012), the use of antigen extracts may be considered problematic from a regulatory standpoint. On the other hand, whereas the immunogenic antigens of viruses such as adenovirus, cytomegalovirus (CMV) or Epstein–Barr virus (EBV) are well described, the antigenic properties of *A. fumigatus* are rather complex and only a few of the hundreds of (glyco)proteins of the fungus reported in the literature have been characterized at a molecular and biochemical level (Latgé, 1999). It has recently been demonstrated that different fungal components are endowed with a distinct, yet overlapping, capacity to activate protective and non-protective T_H_ cell responses in mice and humans (Bozza et al., 2009). In addition, an immunodominant epitope derived from *A. fumigatus* cell wall glucanase Crf1, restricted to three common major histocompatibility complex class II alleles, HLA-DRB1* 03, -04, and -13, has been identified which induces cross-reactive CD4^+^ T_H_1 immunity not only against *A. fumigatus*, but also against *C. albicans* (Stuehler et al., 2011). However, beside the restriction to donors with certain HLA-alleles, it is important to note that the *Aspergillus* cell wall is a highly dynamic structure with the ability to adapt to different environments. Depending on the specific environment, each of the fungal morphotypes may exhibit different biological features, which in turn, can influence the pathogenicity of the pathogen and may result in the differential expression of antigens on its surface. Therefore, the fungal pathogen could easily escape from adoptive immunotherapy using antifungal T cells recognizing only a single antigen, whereas an approach using a fungal extract which includes multiple antigens for generating antifungal T cells might decrease this risk. To this end, the specific advantages and limitations of the use of a lysate from *Aspergillus* isolates and of single or multiple antigens have to be evaluated, and further studies will show which will be the most feasible approach in the clinical setting.

Unfortunately, in the clinical setting, no distinct fungal pathogen can be isolated and characterized in the majority of patients with a suspected invasive fungal infection; this, however, is considered to be a prerequisite for the determination of the antigen in order to generate pathogen-specific T cells. In addition, imaging studies such as computed tomography (CT) scan or the detection of a fungal antigen such as galactomannan, does not prove an infection due to a specific fungal pathogen. Lastly, a considerable number of patients suffer from co-infection with several species or genera of fungi. Therefore, similar to the approach in antiviral immunotherapy using T cell populations that target CMV, EBV, and adenovirus simultaneously (Hanley et al., 2009), the generation of multi-specific T cells which target a variety of different clinically important fungi such as *Aspergillus* spp., *Candida* spp., and Mucormycetes would be of major advantage. We recently reported on an approach of generating multi-specific human antifungal T cells after simultaneous stimulation with cellular extracts of *A. fumigatus*, *C. albicans*, and* Rhizopus oryzae* (Tramsen et al., 2013a). These generated cells consisted of activated memory T_H_1 cells and reproducibly responded with IFN-γ production to a broad-spectrum of medically important fungal pathogens, such as *A. fumigatus*, *A. niger*, *Penicillium chrysogenum*, *C. albicans*, *C. tropicalis*, *M. circinelloides*, *Rhizomucor pusillus*, *Rhizopus oryzae*, *Rhizopus microsporus*, and *Rhizopus microsporus-oligosporus*. Upon re-stimulation, the generated T cells proliferated and enhanced antifungal activity of phagocytes, and showed reduced alloreactivity as compared to the original cell fraction. The ultimate goal would be to generate antifungal T cells against all medically important fungi.

Various antifungal agents have been shown to influence the function of the host immune response. For instance, early studies demonstrated that itraconazole suppresses random movement and chemotaxis of neutrophils (Vuddhakul et al., 1990), and liposomal amphotericin B and amphotericin B-deoxycholate show different immunoregulatory effects on human peripheral blood mononuclear cells (Reyes et al., 2000). In addition, liposomal amphotericin B suppresses specific activity of cytotoxic CD8^+^ T cells in the setting of murine listeriosis (Kretschmar et al., 2001). In contrast, we recently reported that various concentrations of commonly used antifungal compounds such as amphotericin B-deoxycholate, liposomal amphotericin B, fluconazole, voriconazole, posaconazole, and caspofungin did not significantly influence the secretion of IFN-γ and tumor necrosis factor-alpha (TNF-α) by human anti-*Aspergillus* and human anti-*Candida* T_H_1 cells (Tramsen et al., 2013b). The proliferation of these cells was slightly decreased by posaconazole at high concentrations only, which, however, did not reach statistical significance. These data suggest that antifungal T cells can be safely administered together with commonly used antifungal compounds.

### ANTIFUNGAL ACTIVITY OF NATURAL KILLER CELLS

Since natural killer (NK) cells are able to kill tumor cells *in vitro*, there is increasing interest in using NK cells as adoptive immunotherapy against malignancies in HSCT recipients. In contrast to adoptively transferred donor-derived T cells, which are associated with the risk of GvHD, NK cells are usually well tolerated and may even mitigate GvHD (Passweg et al., 2006). In addition to the antitumor effect, NK cells exhibit cytotoxicity against virus-infected cells and activity against bacteria such as *Staphylococcus aureus* and against various parasites (Biron, 1997; Lieke et al., 2004; Small et al., 2008). There is also growing evidence of *in vitro* and animal studies that NK cells play an important role in the host response against fungal pathogens. For example, *in vitro* data demonstrate that NK cells are able to damage *Aspergillus* spp. and *Rhizopus oryzae* (Bouzani et al., 2011; Schmidt et al., 2011, 2012). Importantly, hyphae of both fungi are damaged by both freshly isolated and IL-2 pre-stimulated NK cells, whereas conidia are not affected (Bouzani et al., 2011; Schmidt et al., 2011, 2012). The *in vitro* data on the damage of *Aspergillus* spp. by NK cells are supported by animal studies (Morrison et al., 2003; Park et al., 2009). For example, in neutropenic mice suffering from pulmonary aspergillosis, the depletion of NK cells by antibodies resulted in a greater than twofold increase in mortality and markedly reduced clearance of the pathogen from the lungs (Morrison et al., 2003). Similarly, depletion of NK cells reduced lung IFN-γ levels and subsequently increased fungal load, whereas the transfer of activated NK cells from wild-type, but not from IFN-γ-deficient mice resulted in greater pathogen clearance from the lungs, which supports the importance of functionally active NK cells in the antifungal host response (Park et al., 2009). If further studies evaluating the effect and side effects of adoptively transferred NK cells to an immunocompromised host with invasive fungal infection will demonstrate a benefit, NK cells might become an interesting tool in immunotherapeutic antifungal strategies.

## CONCLUDING REMARKS

Invasive fungal infections, in particular infections due to *Aspergillus*, *Candida*, and Mucormycetes, are still a major cause of morbidity and mortality in HSCT patients. These patients suffer from long lasting defects of the host immune response, and despite the availability of new antifungal compounds, mortality in this patient population is unacceptably high. Over the last decades, our knowledge of the immunopathogenesis of invasive fungal infections has greatly advanced, and the role of different arms of host immunity, such as the roles of phagocytes, T cells and NK cells have been elucidated. Data of *in vitro* experiments and animal studies have provided us with important information to augment host immunity, which resulted in growing interest in the clinical application of immunotherapeutic approaches in HSCT recipients suffering from invasive fungal infections. Although further information obtained from preclinical *in vivo* models is crucial, only clinical trials will ultimately demonstrate the impact of specific cell populations in the prevention or treatment of invasive fungal infections in the transplant recipient. These clinical trials have to address important questions such timing of intervention, type and dosing of adoptively transferred cells, and eligible patients. The latter most likely depends on the host’s unique genetic background, which might have important impact on susceptibility, clinical course, and outcome of invasive fungal infections. In addition, it is important to note that patients suffering from invasive fungal infection are a heterogenous population, not only regarding the underlying pathogen, but also regarding affected organs as well as antifungal pre-treatment. Therefore, it will be difficult to prove a clinical benefit of a specific immunotherapeutic strategy in a sufficiently powered number of patients. In order to design meaningful clinical trials, international, multi-center collaboration is required, which hopefully will improve the outcome in immunocompromised patients suffering from invasive fungal infection.

## Conflict of Interest Statement

Thomas Lehrnbecher is a consultant to Astellas, Gilead, Merck, and Sharp & Dohme, and served at the speakers’ bureau of Astellas, Gilead,Merck, Sharp & Dohme, and Pfizer. All the other authors: none to declare.
